# Supplementary material for: Performance of COVID-19 associated symptoms and temperature checking as a screening tool for SARS-CoV-2 infection
Source: PLoS One. 2021 Sep 17;16(9):e0257450. doi: 10.1371/journal.pone.0257450 (PMC8448301; doi:10.1371/journal.pone.0257450)
Supplement: S1 Table — (DOCX) [file pone.0257450.s001.docx]

**S1 Table. ROC showing the performance of temperature screening for SARS-CoV-2 infection at given age range**

| **Temperature (**^o^C**) cut-point >/=** | **Sensitivity** | **Specificity** | **Correctly classified** | **LR+** | **LR-** |
| --- | --- | --- | --- | --- | --- |
| **1-19 years** | **N=133 (6.7%), ROC area 0.418 [0.278-0.557]** | | |  |  |
| 36.7 | 14.3 | 77.3 | 70.7 | 0.63 | 1.11 |
| 37.3 | 0.00 | 94.1 | 84.2 | 0.00 | 1.06 |
| 37.5 | 0.00 | 95.8 | 85.7 | 0.00 | 1.04 |
| 38.0 | 0.00 | 96.6 | 86.5 | 0.00 | 1.03 |
| 38.2 | 0.00 | 97.5 | 87.2 | 0.00 | 1.03 |
| 39.5 | 0.00 | 99.2 | 88.7 | 0.00 | 1.00 |
| **20-39 years** | **N=1,455 (73.3%), ROC area 0.484 [0.445-0.524]** | | |  |  |
| 36.7 | 23.3 | 75.8 | 66.9 | 0.96 | 1.01 |
| 37.3 | 6.5 | 93.8 | 79.1 | 1.05 | 0.99 |
| 37.5 | 6.1 | 95.5 | 80.5 | 1.37 | 0.98 |
| 37.7 | 5.3 | 96.1 | 80.8 | 1.37 | 0.99 |
| 37.8 | 5.3 | 96.5 | 81.1 | 1.49 | 0.98 |
| 38.0 | 3.3 | 96.9 | 81.1 | 1.04 | 0.99 |
| 38.2 | 2.0 | 97.5 | 81.4 | 0.82 | 1.00 |
| 38.5 | 1.2 | 98.1 | 81.8 | 0.64 | 1.01 |
| 38.7 | 1.2 | 98.6 | 82.2 | 0.87 | 1.00 |
| 39.0 | 0.4 | 99.0 | 82.4 | 0.41 | 1.00 |
| **>39 years** | **N=397 (20.0%), ROC area 0.479 [0.398-0.560]** | | |  |  |
| 36.7 | 26.7 | 78.6 | 68.8 | 1.24 | 0.93 |
| 37.3 | 8.0 | 95.3 | 78.8 | 1.71 | 0.96 |
| 37.5 | 6.7 | 96.0 | 79.1 | 1.65 | 0.97 |
| 37.7 | 4.0 | 96.3 | 78.8 | 1.07 | 0.99 |
| 37.8 | 1.3 | 96.6 | 78.6 | 0.39 | 1.02 |
| 38.0 | 1.3 | 96.9 | 78.8 | 0.43 | 1.02 |
| 38.4 | 0.0 | 98.5 | 79.9 | 0.00 | 1.02 |
| 39.0 | 0.0 | 98.8 | 80.1 | 0.00 | 1.01 |
