# Supplementary material for: Performance of COVID-19 associated symptoms and temperature checking as a screening tool for SARS-CoV-2 infection
Source: PLoS One. 2021 Sep 17;16(9):e0257450. doi: 10.1371/journal.pone.0257450 (PMC8448301; doi:10.1371/journal.pone.0257450)
Supplement: S2 Table — (DOCX) [file pone.0257450.s002.docx]

**S2 Table. Proportion of participants meeting the Tamale Teaching Hospital, Ghana; updated case definition (July 2020)**

|  |  |  | **SARS-CoV-2 by PCR** | |  |
| --- | --- | --- | --- | --- | --- |
|  | **Case definition** | **Total** | **Negative** | **Positive** |  |
|  |  | **N** | **n (%)** | **n (%)** | **χ^2^ (p-value)** |
|  | **Clinical criteria 1: Any two of; Fever, Cough, Sneezing, sore throat, Runny nose (N=1963)** | | | | |
|  | Did not meet criterion | 1710 | 1437 (84.0) | 273 (16.0) | 7.62 (0.006) |
|  | Met criterion | 253 | 195 (77.1) | 58 (22.9) |  |
|  | **Clinical criteria 2: Any one of: difficulty in breathing, anosmia, ageusia (N=1782)** | | | | |
|  | Did not meet criterion | 1553 | 1312 (84.5) | 241 (15.5) | 48.80 (<0.001) |
|  | Met criterion | 229 | 150 (65.5) | 79 (34.5) |  |
|  | **Tamale Teaching Hospital Criteria 1 or 2 (N=1781)** | | | | |
|  | Did not meet criterion | 1409 | 1197 (85.0) | 212 (15.1) | 39.06 (<0.001) |
|  | Met criterion | 372 | 264 (71.0) | 108 (29.0) |  |
