# Supplementary figures and images for: Performance of COVID-19 associated symptoms and temperature checking as a screening tool for SARS-CoV-2 infection
Source: PLoS One. 2021 Sep 17;16(9):e0257450. doi: 10.1371/journal.pone.0257450 (PMC8448301; doi:10.1371/journal.pone.0257450)

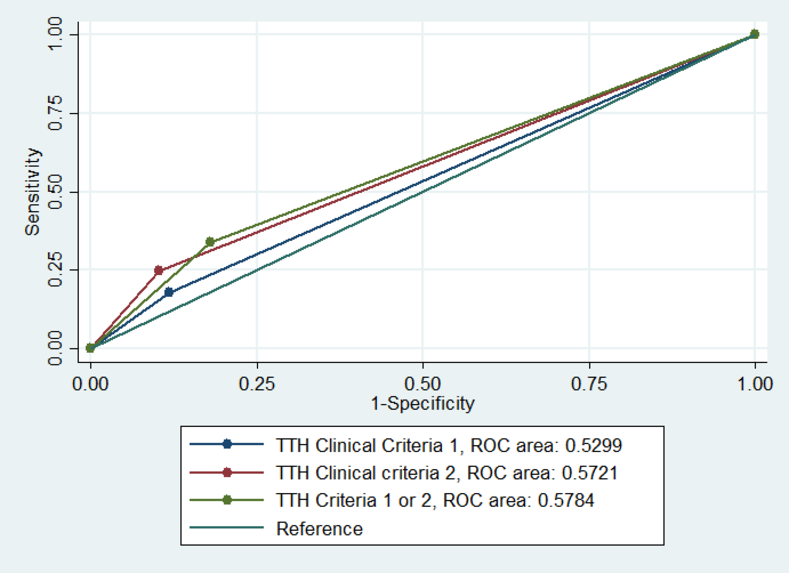

Supplement: S1 Fig — (TIF) [file pone.0257450.s003.tif]
