# Supplementary material for: Performance of COVID-19 associated symptoms and temperature checking as a screening tool for SARS-CoV-2 infection
Source: PLoS One. 2021 Sep 17;16(9):e0257450. doi: 10.1371/journal.pone.0257450 (PMC8448301; doi:10.1371/journal.pone.0257450)
Supplement: S1 Text — (DOCX) [file pone.0257450.s004.docx]

**S1 Text.** **Tamale Teaching Hospital; updated COVID-19 case definition**

| **A Suspected case is any person who:**   1. Meets at least **two of clinical criteria 1**  **OR** 2. Meets **one of clinical criteria 2** **OR** 3. Has radiological evidence of SARS-CoV-2 infection   **Clinical criteria 1 (**Meets at least **two)**   - Fever - Cough - Sneezing - Sore throat - Runny nose   **Clinical Criteria 2 (**Meets at least **one)**   - Difficulty breathing - Loss of smell (Anosmia) - Loss of Taste (Ageusia)   **Diagnostic imaging criteria**  Radiological evidence |
| --- |
