# Supplementary material for: Performance of COVID-19 associated symptoms and temperature checking as a screening tool for SARS-CoV-2 infection
Source: PLoS One. 2021 Sep 17;16(9):e0257450. doi: 10.1371/journal.pone.0257450 (PMC8448301; doi:10.1371/journal.pone.0257450)
Supplement: Attachment — Submitted filename: Response to reviewer.docx [file pone.0257450.s006.docx]

**RE: PONE-D-21-14457R1: Performance of COVID-19 associated symptoms and temperature checking as a screening tool for SARS-CoV-2 infection.**

Thank you for the opportunity to provide a revision to this work.

**Response to reviewer**

We are grateful to the editor and the reviewer for your determination to improve this manuscript. This kind gesture is well appreciated. We are privileged to have you review this manuscript. Below is how we address your comment;

**Comments:**

The paper has been greatly improved with more information on the context of the study and additional analyses, I thank the authors for their response. However, some minor issues remain, mainly on the discussion. I will mention some further below for the authors to consider.
- The analysis of the performance on the temperature screening by age is a nice add-on, but it is not discussed at all. Also, the ‘desirable’ performance is not defined in the main text, and in Supplementary Table 1 the number of patients considered in each age group is not given.

**Response**

Discussion on temperature screening by age has been included in the discussion now. We are grateful for pointing this out to us.

Desirable performance defined: the following was added to the method, statistical analysis, page 8, lines 200-202 ***“ The performance of a screening test is deemed to be desirable if Area Under Curve (AUC) is 0.7 ≥ AUC ≤ 0.8, excellent if 0.8 < AUC ≤ 0.9 and outstanding if AUC is > 90 [37].”***

In Supplementary table 1, the number of patients considered and corresponding percentage in each age group has been provided now.

**Comment**
- The analysis of the performance of the combination of symptoms is very interesting. However, I would consider rephrasing the paragraph ‘Combination of symptoms’, as it is not clearly written. Some key figures would improve the reading. Further, these results are not discussed either. Discussing these results, side by side with the temperature check would improve the paper and strengthen the authors’ result that “The use of fever alone or other symptoms individually [or in combination] as a screening tool for SARS-CoV-2 infection is not worthwhile based on ROC analysis”. Also, it could be noted that the case definition of the Tamale hospital (criteria 1 or 2) led to quite a lot of missed cases

**Response**

Paragraph on combination of symptoms was rephrased and re-written. Some key figures were included to improve the reading. Page 14 to 15, lines 268 - 293 shows this modification.

Some key figures to improve reading: supplemental figures 1 and 2 had been moved to main manuscript and named Figures 3 and 4 respectively. Also, a new figure displaying the three ROC curves for clinical criteria 1, 2 and combination of 1 or 2 had been included as supplemental figure 1.

Also, it was noted in the results section, page 15, lines 291 – 293 that, the case definition of the Tamale hospital (criteria 1 or 2) led to quite a lot of missed cases. ***“The combination of symptoms as exemplified by clinical criteria 1 or 2 missed 66.3% of all positive cases.”***

Discussion section has been updated to include reviewer suggestions.

As part of the update on the discussions and to address reviewer comment, the following publications were cited and referenced appropriately.

- Mandrekar JN. Receiver operating characteristic curve in diagnostic test assessment. Journal of Thoracic Oncology. 2010;5:1315–6.
- Bielecki M, Crameri GAG, Schlagenhauf P, Buehrer TW, Deuel JW. Body temperature screening to identify SARS-CoV-2 infected young adult travellers is ineffective. Travel Med Infect Dis. 2020;37:101832. doi:10.1016/j.tmaid.2020.101832.
- Wright WF, Mackowiak PA. Why Temperature Screening for Coronavirus Disease 2019 With Noncontact Infrared Thermometers Does Not Work. Open Forum Infectious Diseases. 2021;8. doi:10.1093/ofid/ofaa603.
- Roland LT, Gurrola JG, Loftus PA, Cheung SW, Chang JL. Smell and taste symptom-based predictive model for COVID-19 diagnosis. International Forum of Allergy & Rhinology. 2020;10:832–8. doi:10.1002/alr.22602.
