# Supplementary material for: Performance of COVID-19 associated symptoms and temperature checking as a screening tool for SARS-CoV-2 infection
Source: PLoS One. 2021 Sep 17;16(9):e0257450. doi: 10.1371/journal.pone.0257450 (PMC8448301; doi:10.1371/journal.pone.0257450)
Supplement: Attachment — Submitted filename: response to reviewer comment.docx [file pone.0257450.s005.docx]

**30^th^ June, 2021**

**The editor**

**PLOS one**

**Dear Editor,**

**Response to Reviewers:** Performance of COVID-19 associated symptoms and temperature checking as a screening tool for SARS-CoV-2 infection

Thank you for the opportunity and improving the quality of this manuscript. Below is how we addressed the comments of the reviewers.

**Journal/Editorial requirement**

- The data set has been made public and can be found here <https://osf.io/fz82k/>
- Also the map was changed using suggested resources

**Reviewer #1:** 

**comment**
- While the usefulness of temperature and other symptoms in COVID-19 screening is a key question, it is important acknowledge that other studies have been conducted, and replace the study in the context of the current literature (e.g. among others, a study in Australia [https://onlinelibrary.wiley.com/doi/10.1111/1742-6723.13578](https://onlinelibrary.wiley.com/doi/10.1111/1742-6723.13578) for temperature check, and a meta-analysis [https://gut.bmj.com/content/70/4/806#ref-1](https://gut.bmj.com/content/70/4/806#ref-1) for loss of smell/taste).

**Response**

**Thank you for the suggestions which significantly improved the manuscript. The abstract and introduction were updated with findings from current literature including the suggested literature to reflect the context**

**comments**
- A more in-depth exploration of the dataset could strengthen the manuscript. For example: is the temperature screening performing differently given the age of the patient? Would other symptoms be interesting to consider in the screening? Would a screening of a combination of symptoms perform better than individual ones? Also, it would be further interesting to place this study in a global context and see if there are any key differences in terms of disease presentation and/or prevalence of different symptoms.

**Response**

**Thank you very much, suggestions were carried out and a more in-depth exploration of the dataset was carried out. The performance of the temperature check at varying age groups were carried out and shown in supplement table 1. Also, a combination of symptoms such as the case definition used for screening in the Tamale teaching Hospital, Ghana attached as supplement table 2 was explored. A section on the combination of symptoms was added to the results and the performance of the combination of symptoms added as table 5.**

**Minor comments:**

**comment**
- In the abstract, the sentence: ‘The likelihood of a positive test result for SARS-CoV-2 was 5.9 [3.9 – 8.8] for loss of sense of smell and 5.9 [3.8 – 9.3] for loss of sense of taste.’ is unclear. A likelihood should be a probability, here it seems to be a relative risk of presenting with the symptoms when being positive, compared to when being negative. It would be better to use the formulation from page 10, which is well written.

**Response:** **Thank you very much, your suggestion was accepted and the formulation in page 10 was used as shown in the abstract, results section lines 56-57**

**Comment**
- In the methods, page 6, it would be useful to see the CT threshold used to declare the RT-PCR as positive.

**Response:**

**The following was included in the method section of the manuscript to address your suggestion. “*Samples with SARS-CoV-2 RT PCR cycle threshold (CT) value under 40 were considered positive”***

**Comment**
- In the methods, page 6, the term ‘Real-time RT-PCR’ is redundant.

**Response:**

**Thank you, “Real-time” was deleted**

**Comment**
- The reporting of the ROC analysis from Table 4 is inaccurate: 4.8% sensitivity corresponds to a temperature cut-point of 37.7, and not 37.8.

**Response**

**Thank you, the error was corrected; 4.8% was changed to 4.2%. the sentence now reads; “For example, using a temperature cut off of 37.8 would only pick 4.2% of positive SARS-CoV-2 infected patients”**

Once again thank you for the suggestions which greatly improved the manuscript

Yours faithfully,

Dr. Benjamin Nuertey
